# Supplementary material for: Analysis of polarimetric satellite measurements suggests stronger cooling due to aerosol-cloud interactions
Source: Nat Commun. 2019 Nov 27;10:5405. doi: 10.1038/s41467-019-13372-2 (PMC6881401; doi:10.1038/s41467-019-13372-2)
Supplement: Supplementary file 1 — Supplementary Information [file 41467_2019_13372_MOESM1_ESM.pdf]

Supplementary information to “Analysis of polarimetric satellite measurements suggests stronger cooling due to aerosol-cloud interactions”

Otto P. Hasekamp[1,*], Edward Gryspeerdt[2] & Johannes Quaas[3]

[1] *SRON Netherlands Institute for Space Research, Sorbonnelaan 2, 3584 CA Utrecht, the Netherlands. Email: O.Hasekamp@sron.nl*

[2] *Space and Atmospheric Physics Group, Imperial College London, London SW7 2AZ, United Kingdom*

[3] *Universität Leipzig, Institute for Meteorology, Stephanstr. 3, D-04103 Leipzig, Germany*

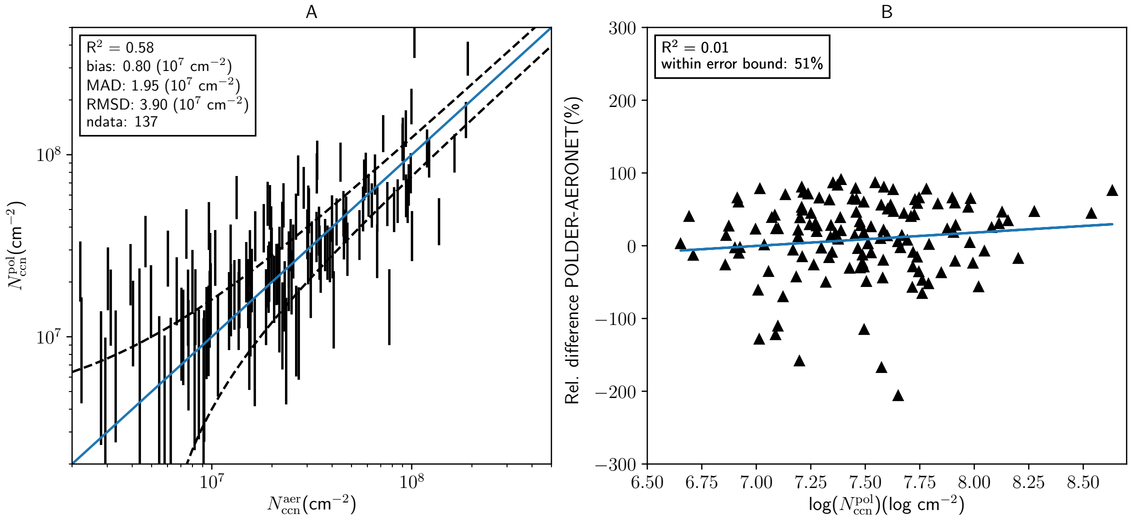

Supplementary Figure 1 - Comparison POLDER CCN column number with AERONET. (A) POLDER retrieved Cloud Condensation Nuclei (CCN) column number ($N_{\mathrm{ccn}}^{\mathrm{pol}}$) versus AERONET $N_{\mathrm{ccn}}^{\mathrm{aer}}$. AERONET data within the POLDER grid cell (1° by 1°) and taken on the same day have been used. The blue line shows the identity line. Error bars show (1-σ) uncertainty found from synthetic experiment. Data within the dashed lines have a difference smaller than ($0.20 \cdot N_{\mathrm{ccn}} + 4 \cdot 10^6$). (B) Relative difference between POLDER and AERONET ((N_{ccn}^{pol}-N_{ccn}^{aer})/N_{ccn}^{pol})*100) as function of log(($N_{\mathrm{ccn}}^{\mathrm{pol}}$)). The blue line shows the linear regression.

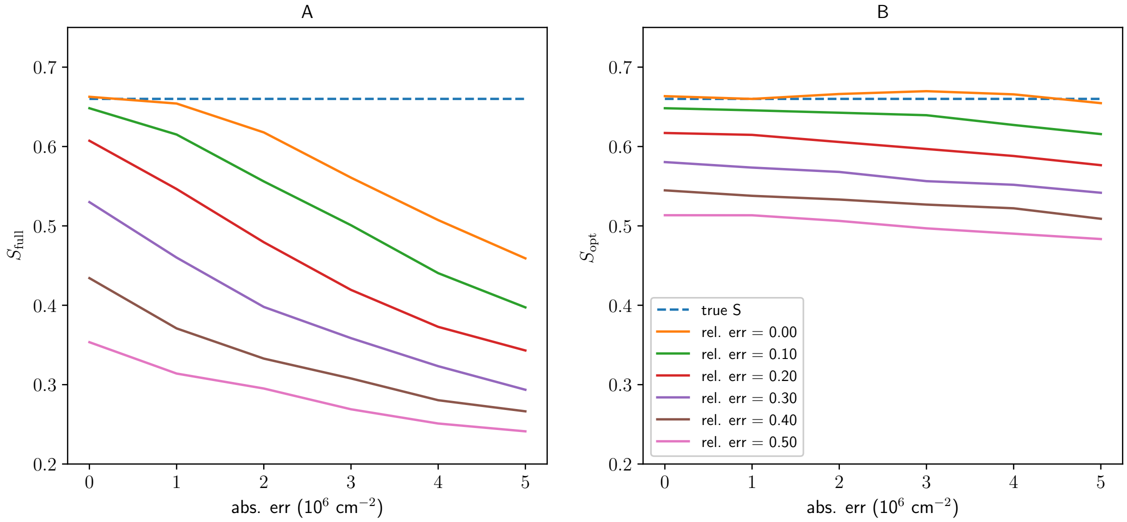

Supplementary Figure 2 - Effect of Measurement Uncertainties. Derived susceptibilities from simulated measurements of Cloud Condensation Nuclei (CCN) column number (N_{ccn}) and cloud droplet number concentration (N_{d}) as function of absolute error on N_{ccn} for different values of the relative error. Panel A shows results for S_{full} derived using the full range of N_{ccn} and panel B shows results for S_{opt} using only $N_{\mathrm{ccn}} > 10^7 \mathrm{cm}^{-2}$. Synthetic retrievals and comparison to AERONET points out that $(0.20 \cdot N_{\mathrm{ccn}} + 4 \cdot 10^6)$ is a realistic error estimate. The true susceptibility for the simulated measurements is S=0.66.
